# Flexible Multiscale Pore Hybrid Self-Powered Sensor for Heart Sound Detection

**DOI:** 10.3390/s21134508

**Published:** 2021-06-30

**Authors:** Boyan Liu, Liuyang Han, Lyuming Pan, Hongzheng Li, Jingjing Zhao, Ying Dong, Xiaohao Wang

**Affiliations:** 1 Tsinghua Shenzhen International Graduate School, Tsinghua University, Shenzhen 518055, China; liuby18@mails.tsinghua.edu.cn (B.L.); hanly18@tsinghua.org.cn (L.H.); plm14@tsinghua.org.cn (L.P.); li-hz18@mails.tsinghua.edu.cn (H.L.); jjzhao2017@sz.tsinghua.edu.cn (J.Z.); 2 Tsinghua-Berkeley Shenzhen Institute, Tsinghua University, Shenzhen 518055, China

**Keywords:** flexible sensor, electrospinning, piezoelectric polymer, heart sound detection, hybrid self-powered

## Abstract

This research introduces an idea of producing both nanoscale and microscale pores in piezoelectric material, and combining the properties of the molecular β-phase dipoles in ferroelectric material and the space charge dipoles in order to increase the sensitivity of the sensor and modulate the response frequency bandwidth of the material. Based on this idea, a bi-nano-micro porous dual ferro-electret hybrid self-powered flexible heart sound detection sensor is proposed. Acid etching and electrospinning were the fabrication processes used to produce a piezoelectric film with nanoscale and microscale pores, and corona poling was used for air ionization to produce an electret effect. In this paper, the manufacturing process of the sensor is introduced, and the effect of the porous structure and corona poling on improving the performance of the sensor is discussed. The proposed flexible sensor has an equivalent piezoelectric coefficient d_33_ of 3312 pC/N, which is much larger than the piezoelectric coefficient of the common piezoelectric materials. Experiments were carried out to verify the function of the flexible sensor together with the SS17L heart sound sensor (BIOPAC, Goleta, CA, USA) as a reference. The test results demonstrated its practical application for wearable heart sound detection and the potential for heart disease detection. The proposed flexible sensor in this paper could realize batch production, and has the advantages of flexibility, low production cost and a short processing time compared with the existing heart sound detection sensors.

## 1. Introduction

Currently, a variety of flexible sensors have been developed to detect physiological signals of the human body in a wearable way. Heart sounds carry a lot of information about the cardiovascular system. Radar, lasers and accelerometers are ways to detect heart sounds, but they are not flexible and wearable due to their poor anti-jamming ability and heavy weight [1,2,3,4]. Flexible sensors based on resistance and capacity need an external power supply, and triboelectric sensors are greatly affected by external humidity, but sensors with a piezoelectric effect not only avoid an external power supply but also have a quick response with a fast charge transfer speed. Thus they are more suitable for measuring high-frequency dynamic signals such as heart sounds [5,6,7,8]. To date, most reported sensors for heart sound detection rely on piezoelectric materials such as PZT. It is well known that PZT has excellent piezoelectric properties with a piezoelectric coefficient of about 200 pC/N, but it is too brittle to be integrated into flexible sensors [9]. However, the piezoelectric coefficient of the flexible ferroelectric polymer poly (vinylidene fluoride) (PVDF) is only about 20 pC/N [10]. In addition, flexible materials generally have a smaller Young’s modulus than brittle materials, so the detecting frequency bandwidth is limited [11,12,13]. Heart sounds are a kind of signal with low energy and high frequency compared with other physiological signals such as pulse waves and respiration. Thus it is very important to improve the sensitivity of the sensor. Considering the shortcomings of the existing heart sound detection research, it is necessary to fabricate self-powered flexible sensors for wearable heart sound detection. Meanwhile, it is necessary to design the sensor fabrication process so as to realize low-cost batch processing.

There are many ways to improve the sensitivity, such as the fiber structure’s [14,15,16,17] micropores. The fiber structure has a large aspect ratio, which makes it easier to deform, and produces a higher signal output [18,19,20]. Fiber structures can be made by many techniques, such as electrospinning [21], tensiling [19] and phase separation [22]. Electrospinning is a low-cost process used to fabricate fiber structures on a large scale, while the lapping of the electrospinning fibers forms micropores. A microporous structure is a kind of microstructure that can change the Young’s modulus of the material, thus changing the sensor’s performance. Rybyanets et al. proposed that, compared with the dense film, porous piezoelectric materials have better hydrostatic performance and their acoustic impedance is low, so by introducing controllable pores, the acoustic performance and ultrasonic response can be significantly improved [23]. Khanbareh et al. prepared a porous PZT–PU composite structure with a piezoelectric coefficient of 165 mV/N through the chemical reaction of water and polyurethane (PU) [24]. Moreover, porous electrets are a kind of flexible material showing excellent features of ferroelectricity after corona poling [25]. Kachroudi et al. used a porous polydimethylsiloxane (PDMS) piezoelectric electret to achieve a stable sensitivity of 385 mV/g [26], but its piezoelectric features were due to the arrangement of space charges, which is different from the traditional ferroelectric materials, where the piezoelectric feature is due to non-centrosymmetric crystal structure deformation caused by dipoles. At the same time, the researchers found that as the pore size decreased, the performance improved [27]. There are many ways to create pores, such as gas evaporation [24], phase separation [28] and etching [29,30]. Ways of creating pores in nanofibers have also been studied [27]. Using ZnO nanoparticles to etch the piezoelectric materials can obtain the pores of the same size, and the processing speed is controllable [29]. Combining the microstructure of fibers and pores together, this paper proposes a flexible sensor for heart sound detection. The sensor adopts a sensitive three-layer structure composed of two non-polar material PDMS films as the electret layers, and a porous polar material polyvinylidene fluoride trifluoroethylene (P(VDF-TrFE)) film as the sandwiched piezoelectric induction layer.

## 2. Fabrication and Characterization

### 2.1. Fabrication of the Pressure Responsive Film

**Figure 1:**
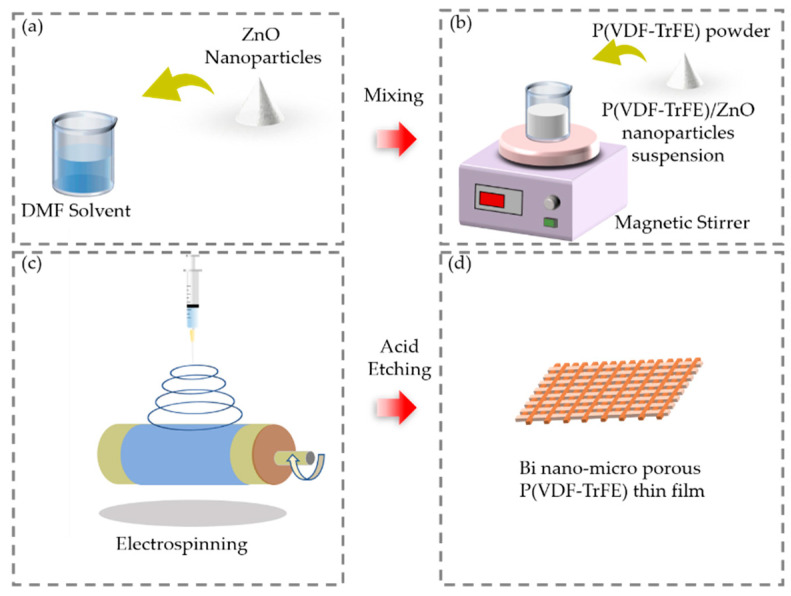
Schematic of the process for producing the pressure-responsive film by electrospinning and acid solution etching. (**a**) Mixing the ZnO nanoparticles; (**b**) dissolving the P(VDF-TrFE) powder. (**c**) Conceptual structure of electrospinning. (**d**) Responsive film after acid etching.

The fabrication process of the bi-nano-micro porous P(VDF-TrFE) film is illustrated in detail, as shown in Figure 1. The P(VDF-TrFE) copolymer was selected instead of PVDF as it has only one ferroelectric crystalline structure (β-phase) at an ambient temperature far below the Curie temperature (above which the polarization and piezoelectric effects of the material will disappear). Electrospinning and hydrogen chloride (HCl) acid solution etching were used to obtain a bi-nano-micro porous film. Electrospinning can prepare uniform and continuous nanofibers on a large scale, and the diameter of the fibers is generally between 100 nm and 10 μm.

Due to the synergistic effect of high voltage and tensile force in the process of electrospinning, the fibers have piezoelectric properties, and the piezoelectric coefficient is perpendicular to the length. The microstructure in piezoelectric fibers can change the intrinsic piezoelectric properties of the fibers such as the Young’s modulus so as to change the piezoelectric coefficient. Abolhasani et al. proved that the sensor’s performance increases with an increase in the porosity of the electrospinning piezoelectric fibers by both simulation and experimental results [28]. This is because the dielectric constant and elastic modulus of the piezoelectric fibers decrease with the increase in porosity, and the fibers have larger deformation under the same pressure. The porous structure can be produced by a variety of fabrication processes, such as freeze-drying [31] and etching [29,30]. Cha et al. proved by both simulation and experimental results that the smaller the porous structure, the larger the output voltage [27]. This is because the nanoscale porous structure limited the stress to the maximum along the Z axis, which is called the geometric strain confining effect. Pores with a diameter of nanometers can be produced by etching ZnO nanoparticles, and the experimental process is controllable. Introducing ZnO nanoparticles to the P(VDF-TrFE) nanofiber film has two roles as well: creating nanoscale pores and seeding the formation of the β-phase [29]. Mao et al. found through experimental results that when the mass fraction of ZnO nanoparticles was 50%, the content of piezoelectric β-phase was the highest [29].

Firstly, acetone (99.9%) and N, N-dimethylformamide (DMF, anhydrous, 99.8%) were mixed in a volume ratio of 3:2 as the organic solvent. Next, 50 wt% ZnO nanoparticles (30–40 nm, Aladdin) were dispersed in the organic solvent through an ultrasonication process for 30 min, as shown in Figure 1a. After that, P(VDF-TrFE) powder (75/25 mol%) was evenly dissolved in this solution by continuous magnetic stirring for 3 h, as shown in Figure 1b [32]. The obtained suspension was then made into a film by electrospinning, as shown in Figure 1c. The syringe needle for electrospinning was selected as 0.34 mm and the syringe flow rate was selected as 2 mL/h to produce nanofibers with an average diameter of 250 nm [33]. The film collector comprised a rotating cylinder with a diameter of 20 cm. The cylinder was wrapped with a 12-μm thick aluminum film. The roller was grounded, and the syringe was connected to the positive electrode with a high voltage of 15 kV [34]. The distance between the roller and the syringe was set as 12 cm. Subsequently, the film was treated in an ultrasonic bath in concentrated HCl (37 wt%) for 3 h to fully etch away the ZnO nanoparticles, and then sonicated with deionized (DI) water for 1 h to fully remove the Zn^2+^ that remained in the nanofibers, as shown in Figure 1d.

### 2.2. Fabrication of the Flexible Sensor

**Figure 2:**
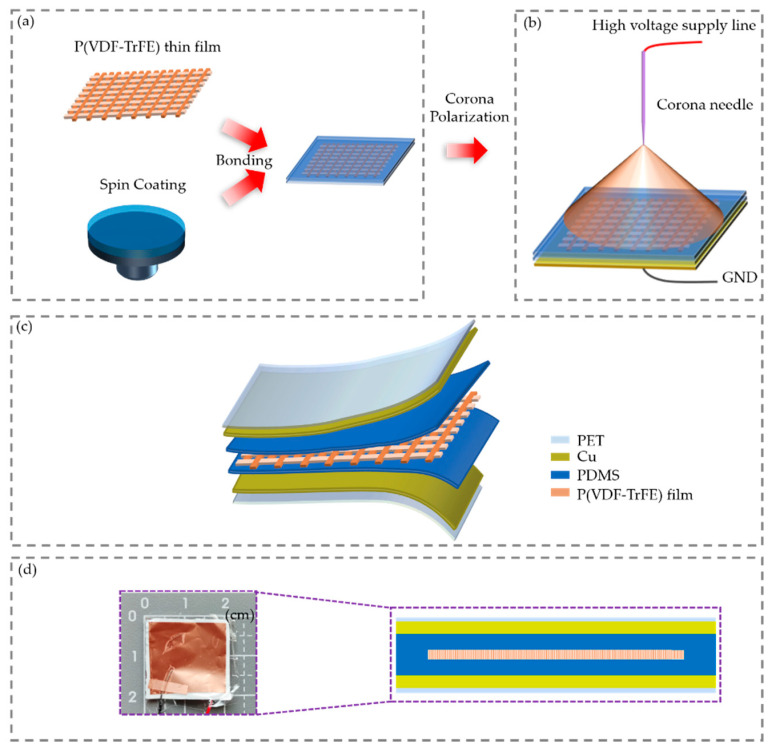
Schematic procedure for fabricating the flexible sensor. (**a**) PDMS sealing as the protective layers and electret layers. (**b**) Corona polarization. (**c**) Diagram of the sensor. (**d**) A sample measuring 2 × 2 cm^2^ (left) and a cross-sectional diagram of the sensor’s structure (right).

The fabrication process of the flexible sensor based on the bi-nano-micro porous P(VDF-TrFE) film is illustrated in Figure 2. PDMS is an excellent electret material [31] and can bond together through molecule force to achieve a sealing effect [35]. The P(VDF-TrFE) ferroelectric sensing layer with a bi-nano-micro porous structure was encapsulated by 2 PDMS electret layers to provide the condition of an airtight environment after corona polarization so as to prevent loss of the injected spacer charges [36]. To prepare the PDMS layers, PDMS was first mixed with a crosslinking agent at the ratio of 10:1. After vacuuming, the spin coater was set at 4000 r/min to spin PDMS onto a 5.08-cm silicon wafer and then the coated wafer was cured at 80 °C in a dryer for 30 min. Because the thickness of the spin-coated PDMS was only several 10 of microns, it was very difficult to remove the PDMS directly after curing. In order to take the film off unscathed, the PDMS and the silicon wafer were isolated by a 25 μm thick fluorinated ethylene propylene (FEP) film. FEP has a smooth surface and low surface energy [37]. Both the PDMS and FEP films were removed from the silicon wafer and then treated with oxygen plasma for 3 min. The P(VDF-TrFE) film was put between 2 pieces of PDMS and then this sandwich structure was put into a dryer at 80 °C for 24 h, as shown in Figure 2a. By evaporating 10 nm chromium as the adhesive layer and 500 nm copper as the electrode on one side of the PDMS surface. The electrode of the film was grounded, and the distance between the needle and the film was set as 3 cm. An electric field of −15 kV was applied to the needle for corona poling for 5 min, as shown in Figure 2b [38]. Pores with a spacing of 5–15 μm are naturally formed by the lapping of the electrospinning nanofibers [39,40]. Through corona polarization, the air in the pores between fibers is ionized to form positive and negative gas charges, which are respectively captured by the upper and lower PDMS electrets to form the space charge dipole moments. After corona poling, a copper tape was affixed to the other side of the PDMS as the mechanical support and the other electrode of the sensor. PET tape with a thickness of 6 μm was used to protect the electrode and separate the sensor from the human body, as well as to achieve an encapsulation effect, as shown in Figure 2c. PET is a smooth and shiny polymer with excellent physical and mechanical properties in a wide temperature range, as well as excellent electrical insulation at high temperatures and high frequencies. In this process, the nanofiber piezoelectric film with an area of 10 × 40 cm^2^ and a thickness of 40 μm was obtained at one time within 3 h based on the electrospinning process. The film was then cut into 100 pieces, each of which was used as a pressure responsive film of a sensor. This fabrication technique can be used for mass production of the sensors. Figure 2d (left) shows a sample with an effective sensing area of 2 × 2 cm^2^, and Figure 2d (right) shows a cross-sectional diagram of the sensor’s structure. The thickness of the sensor was 50–100 μm and had very good flexibility.

### 2.3. Characterization

PVDF and its copolymers usually can be identified by X-ray diffraction analysis (XRD) and Fourier transform infrared spectroscopy (FTIR). The infrared spectrum is very sensitive to the dipole orientation, thus explaining the spontaneous dipole switching during electrospinning.

**Figure 3:**
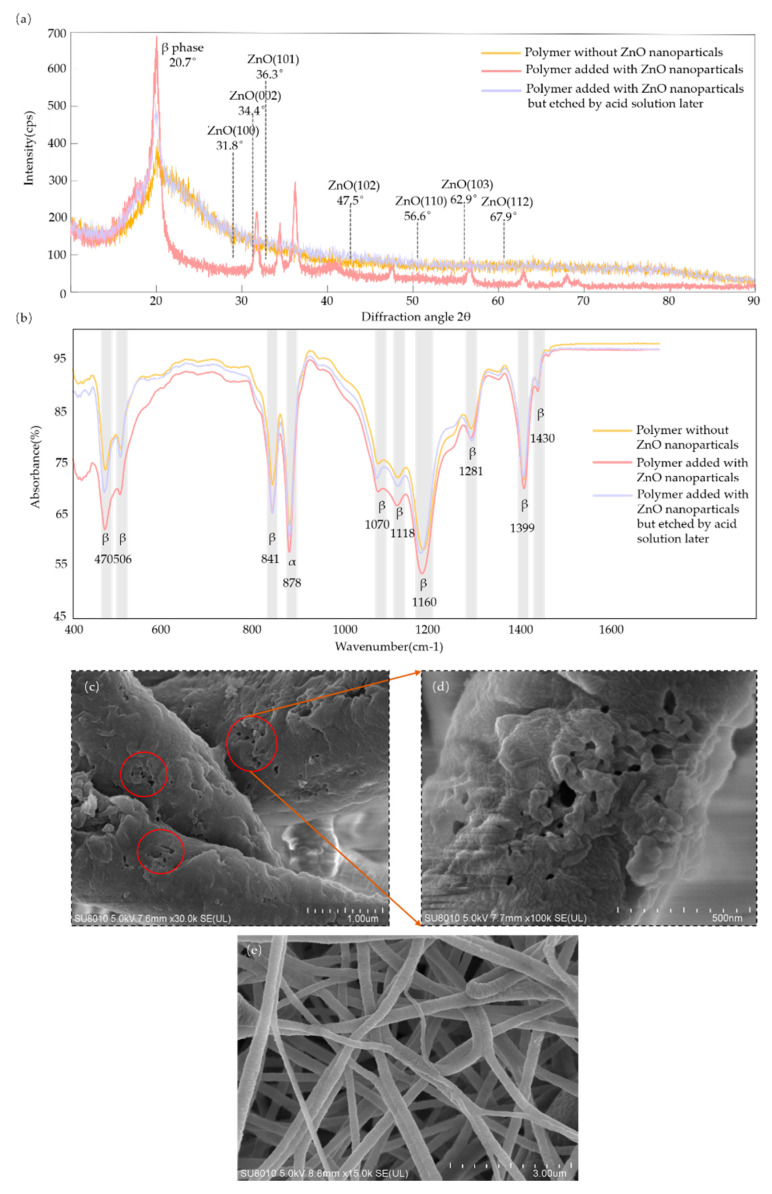
Schematic of analysis of the characteristics: (**a**) XRD spectra; (**b**) FTIR spectra of three kinds of P(VDF-TrFE) polymer film; (**c**) SEM image of nanoscale pores in the fibers (measuring scale in 1 micron). (**d**) Enlarged view of the nanoscale pores (measuring scale in 500 nm). (**e**) Overall morphology of the electrospinning fibers (measuring scale in 3 microns).

The XRD data in Figure 3a show that wurtzite ZnO crystals of (100), (002), (101), (102), (110), (103) and (112) existed in the polymer with added ZnO nanoparticles but were not found in the polymer without ZnO nanoparticles or in the polymer with added ZnO nanoparticles but etched by the acid solution later [41,42]. This means that the ZnO nanoparticles were completely removed via chemical etching. The addition of nanoscale ZnO nanoparticles could increase the crystal content of the β-phase (2θ = 20.7°) through a comparison of the polymer without ZnO nanoparticles and the polymer with added ZnO nanoparticles [43]. The content of the β-phase of the polymer with added ZnO nanoparticles but etched by the acid solution later was higher than that of the polymer without ZnO nanoparticles, which means that acid etching did not significantly weaken the crystal content of the β-phase.

FTIR spectrum analysis is an analytical technique which provides information about the chemical bonds or molecular structure of a material. As shown in Figure 3b, the FTIR spectrum confirmed the crystal content of the β-phase at wavenumbers of 470 cm^−1^, 506 cm^−1^, 841 cm^−1^, 1070 cm^−1^, 1118 cm^−1^, 1166 cm^−1^, 1281cm^−1^, 1399 cm^−1^ and 1430 cm^−1^, and of the α-phase at 878 cm^−1^ [44,45,46]. It showed a similar relationship to that in Figure 3a, where the content of the β-phase of the polymer with added ZnO nanoparticles but etched by the acid solution later was higher than that of the polymer without ZnO nanoparticles but lower than that of the polymer with added ZnO nanoparticles. As shown in Figure 3c–e, nanofibers and nanoscale pores in the fibers were observed by scanning electron microscopy (SEM). According to the ratio scale, the pore size in the fibers was about 30 nm, which corresponded to the size of the ZnO nanoparticles. The diameter of the nanofiber was almost 500 nm according to the ratio scale.

## 3. Working Mechanism

**Figure 4:**
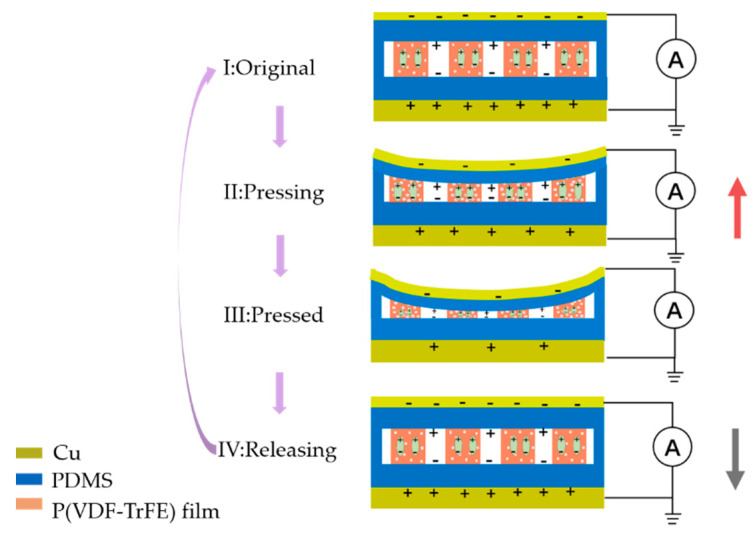
Schematic of the working principle of the flexible sensor.

Mandal et al. elucidated the preferential orientation of molecular dipoles (CH2/CF2-dipoles) through the experimental results of polarized FTIR spectroscopic techniques [47]. The molecular dipoles were oriented along the fiber and generated piezoelectric energy power at a perpendicular orientation to the fiber. The charge distribution in the electrospinning film was different from that of coating polymers (d33 ~ 2d31). Due to the anisotropy of the pores and the low symmetry of the charges, high sensitivity was mainly found along the thickness direction (determined by d33), which was usually two orders larger than that in the horizontal direction (determined by d31 and d32). In the process of corona poling, the air in the sealed cavity was ionized to produce the same amount of positive and negative charges. Under the electric field, positive and negative charges then move up and down to the electret PDMS layers, respectively, forming a large number of dipoles. Figure 4 shows a schematic of the working mechanism of the sensor in short-circuit mode under a cycle of dynamic stimulus. In the initial state (Figure 4, I), molecular dipoles in the ferroelectric material and the real charge potentials on the electret film form an electric field balance, and there is no electric response. When external pressure is applied to compress the sensor (Figure 4, II), the dipole moment changes and the electric field balance is destroyed, and the charges are redistributed to reach a new balanced state, which will form a positive current in the external circuit. Until a new balance is established, there is no charge transfer (Figure 4, III). When the pressure is released, the sensor returns to its original state due to its elasticity, generating a current in the opposite direction in the external circuit (Figure 4, IV) [36,48,49]. This exhibits ferro-electret hybrid self-powered piezoelectric sensing characteristics.

## 4. Results and Discussion

Before the sensor was applied to a specific scenario, its inherent performance was tested. With the microscale and nanoscale pore structures and the corona polarization treatment as variables, four different sample sensors were prepared for contrast experiments to test the effects of multiscale pores and corona poling on sensor performance through comparing the piezoelectric coefficients. The four sensors are listed as: a commercial piezoelectric sensor (MEAS, USA); electrospinning sensor samples with microscale pores without treatment with ZnO nanoparticles and corona polarization (electrospun piezoelectric sensor); electrospinning sensor samples treated with microscale pores and treated with corona polarization, but without ZnO nanoparticles (electrospun hybrid self-powered sensor); and electrospinning sensor samples with both microscale and nanoscale pores after treatment with ZnO nanoparticles and corona polarization (multiscale pore hybrid self-powered sensor).

### 4.1. Equivalent Piezoelectric Coefficient (d_33_)

**Figure 5:**
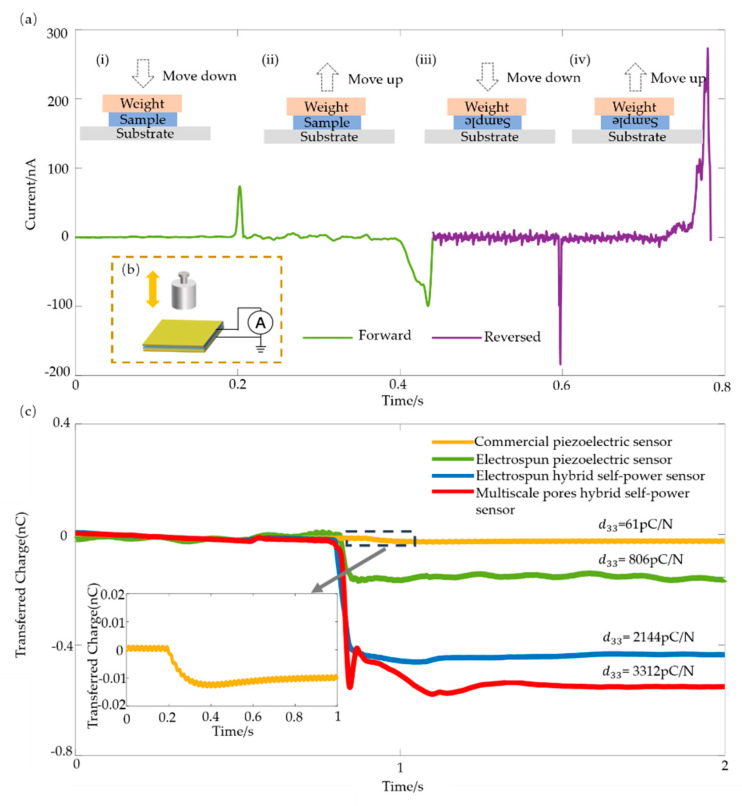
Schematic of the piezoelectrical effect and piezoelectric coefficient d_33_ test. (**a**) Short-circuit current responses of the sensors during one cycle of pressing and releasing with the forward and reversed electrical connections. (**b**) Comparison of the transferred charge of the four sensors with different fabrication processes and microstructures during the test of d_33_. (**c**) The transferred charge and the corresponding piezoelectric coefficient for sensors with different structures under the same mechanical stimulation (inset is the enlarged view of the dashed frame).

The piezoelectric effect produces a directional charge: when the reversal film has a negative connection, the output voltage also reverses. In order to confirm that the output signal was caused by the piezoelectric effect and the electret effect, rather than another artificial charge or interference, we performed the polarity switch test. Figure 5a illustrates the results of verifying the piezoelectric effect of the sensor. Figure 5b shows the schematic of the weight moving method. Through the process of adding and taking off (Figure 5a(i,ii)) the weight, the sensor had a forward and reversed short-circuit current response. Through operation with the electric connection of the sensor reversed (Figure 5a(iii,iv)), the output was also reversed. This verified the piezoelectric characteristics of the sensor and confirmed that the signal generated in one cycle of pressing and releasing was induced by mechanical stimuli rather than triboelectricity or artifacts. The difference in the current peak value in the process of forward and reverse connections was due to the asymmetry of the electrodes. The two electrodes were copper, fabricated by electron beam evaporation and copper in a tape.

The piezoelectric coefficient d_33_ is an important factor for measuring the piezoelectricity of a material. The d_33_ coefficient can be tested using the weight moving method as well. A weight of m = 20 g was placed on the surface of the sample sensor, and a data acquisition module (NI USB-6009) was used to record the data. The calculation formula is:

d_33_ = σ/P = σA/PA = Q/F, (1)

where σ is the charge density produced under the external pressure P, A is the area of the sensor, Q is the induced charge of the sensor under the applied force F. The transferred charges of the four sensors under a weight are shown in Figure 5c. For commercial piezoelectric sensor, electrospun piezoelectric sensor, electrospun hybrid self-power sensor and multiscale pores hybrid self-power sensor, the calculated piezoelectric coefficient d_33_ was 61 pC/N, 806 pC/N, 2144 pC/N and 3312 pC/N, respectively. The piezoelectric coefficient of the electrospun piezoelectric sensor was 806 pC/N, which is about 13 times higher than that of the commercial piezoelectric sensor (61 pC/N). This proved that the presence of microscale holes in the piezoelectric material improved the piezoelectric performance of the sensor. The piezoelectric coefficient of the electrospun hybrid self-powered sensor was 2144 pC/N, which is 2.7 times higher than that of the electrospun piezoelectric sensor. This is because the electrospinning film treated with corona polarization superpositions the piezoelectric effect of the ferroelectric material with the electret effect. The piezoelectric coefficient of the multiscale pore hybrid self-powered sensor was 3312 pC/N, which is 1.5 times that of the electrospun hybrid self-powered sensor. It can be considered that the nanoscale pore structure in the fiber improved the performance of the sensor.

### 4.2. Dynamic Response Characteristics

**Figure 6:**
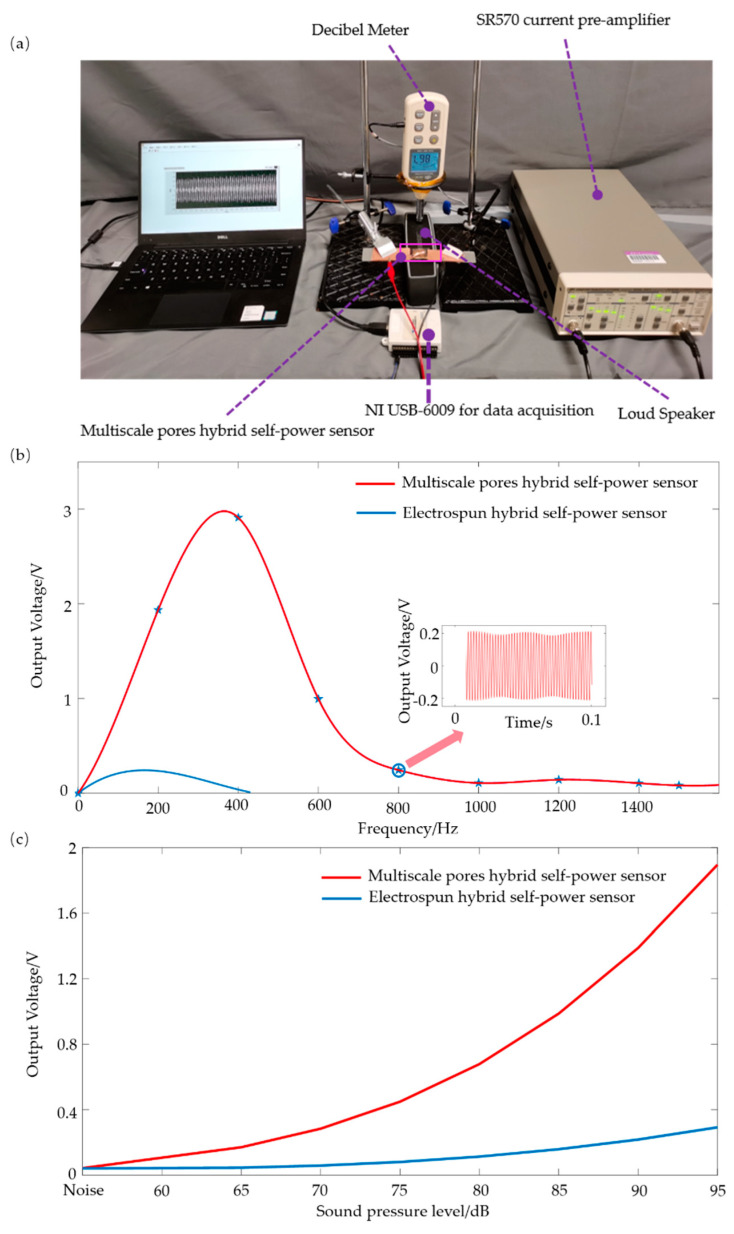
Schematic of the dynamic response of the flexible sensor. (**a**) Testing the dynamic stimulus system. (**b**) Responses of the sensors under changeable sound frequency (SPL-95 dB). (**c**) Responses of the sensors under a change able SPL (frequency-200 Hz).

For the purpose of studying the response characteristics of the flexible sensors, the direct contact method was not applicable, because the mass of the flexible sensor was much smaller than that of the exciter [50]. Heart sounds comprise a variety of different pure tone signals, namely a number of superimposed sinusoidal signals. In order to calibrate the frequency characteristic performance of the flexible sensor, sinusoidal signals generated by a loudspeaker (BOSE) were used. Figure 6a shows a schematic of the dynamic mechanical stimulus test system. The sample was suspended above the loudspeaker, the feedback circuit comprised a data acquisition module (NI USB-6009) and the loudspeaker was controlled by LabVIEW and a decibel meter to generate pure tone signals at different frequencies or different sound pressure levels. The output signal of the flexible sensor was in the scale of nA, and the low-noise current pre-amplifier SR570 (Stanford Research Systems, Sunnyvale, California, USA) was set at 50 nA/V as the amplifier gain to amplify the current into a voltage in the range of −2 V to 2 V. The distance between the loudspeaker and the sensor was set to 1 cm.

Firstly, the response at different frequencies with a constant sound pressure level (SPL) was tested. The sound pressure level was set as SPL = 95 dB and the sound frequency changed in the range of 0–1600 Hz with an interval of 200 Hz. As shown in Figure 6b, the multiscale pore hybrid self-powered sensor had a much wider and higher dynamic stimulus response than the electrospun hybrid self-powered sensor. The latter could only respond to the dynamic stimuli under 400 Hz, while the former could detect stimuli well at around 400 Hz, and the output voltage was higher under the same test conditions. The response at 1000 Hz is shown in the small window in Figure 6b. This indicates that the nanoscale pores played an important role in improving the performance of the sensor. The main energy spectrum of heart sounds consists of the first and second heart sounds, with frequencies ranging from 20 to 200 Hz [51,52]; the flexible sensor exactly can meet the frequency requirements of heart sound detection. The sound frequency was set as 200 Hz and the SPL changes were in the range of 65–95 dB with an interval of 5 dB. Figure 6c shows that the output voltage of the multiscale pore hybrid self-powered sensor was much higher than that of the electrospun hybrid self-powered sensor. The smaller pores may have enhanced the ability to absorb sound, where acoustic energy is transferred more into dynamic energy, leading to less sound reflection [53]. The output voltage was also higher due to the geometric strain limiting effect of the nanoscale pores [28].

**Figure 7:**
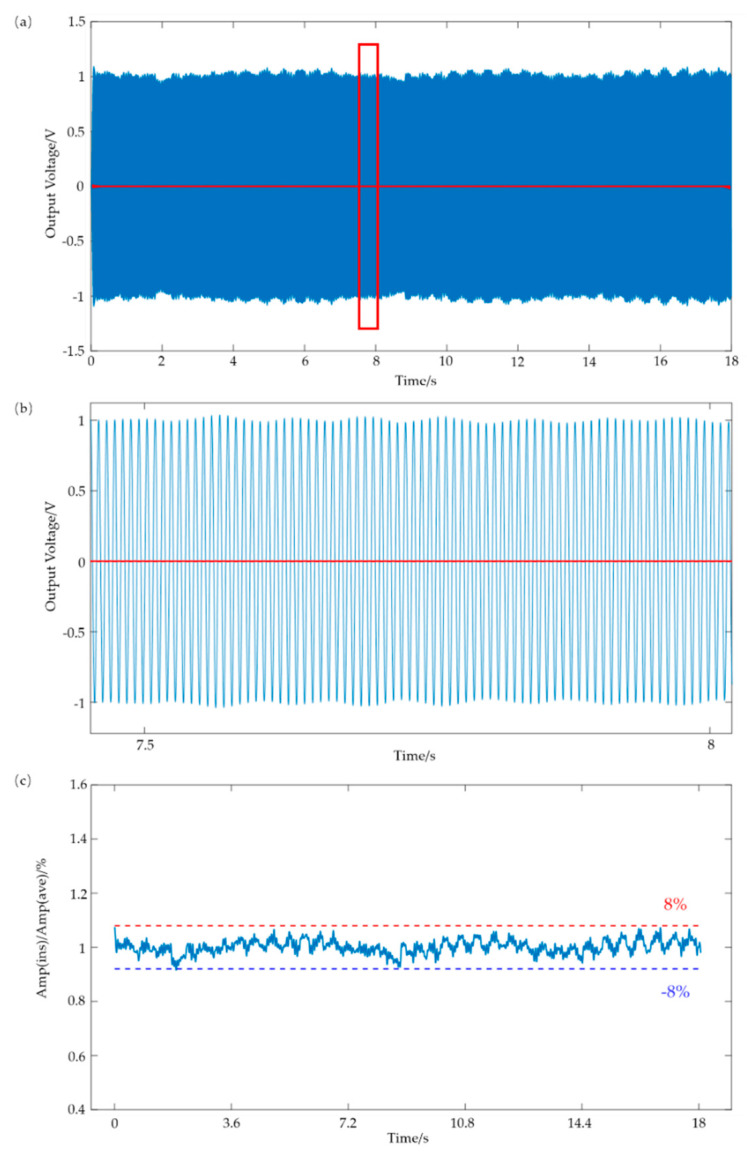
Schematic of the dynamic response of the flexible sensor. (**a**) Output voltage of the sensor versus time under a constant applied dynamic stimulus for 2500 cycles (frequency-140 Hz; SPL-95 dB). (**b**) Enlarged view of the output voltage waveform around 7.5–8 s. (**c**) The drift in Amp (ins) relative to Amp (ave).

For wearable heart sound detection, the long-term stability of the sensor is important. Figure 7a shows the output voltage of the flexible sensor during 2500 cycles within 18 s (frequency: 140 Hz; SPL: 95 dB). Figure 7b is an enlarged view of the red rectangular box in Figure 7a that shows the output’s waveform around 7.5–8 s. The baselines in Figure 7a,b are also shown in red color, and were almost horizontal and only fluctuated within a small range. The instantaneous output voltage (represented as Amp (ins)) fluctuated within 8% and −8% of the average value (represented as Amp (ave)), as shown in Figure 7c.

### 4.3. Pathological Heart sound Detection

**Figure 8:**
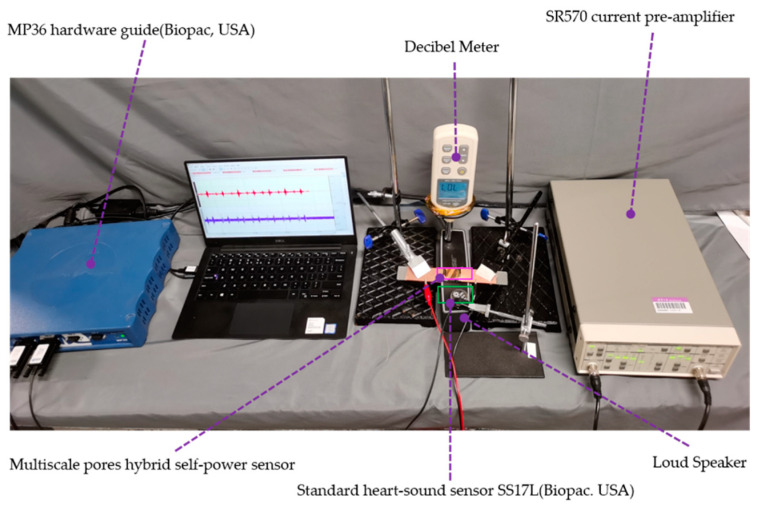
Schematic of the device for comparing the multiscale pore hybrid self-powered sensor and the standard heart sound sensor (SS17L, Biopac, Goleta, CA, USA).

Distinguishing pathological heart sound signals is of great significance for the detection of heart diseases. To confirm that the flexible sensor could distinguish pathological heart sound signals, recorded pathological heart sounds (Medzcool, Youtube, https://youtu.be/uZysrKXHJMM, September 2019) were used as input signals. The experimental platform is shown in Figure 8. The sensor and the standard SS17L heart sound sensor (Biopac, Goleta, CA, USA) were used simultaneously to record the heart sound signals played by the loudspeaker (BOSE), where SS17L was used as the benchmark. The SPL of the loudspeaker was controlled to be 60 dB. The amplifier gain of the low-noise current pre-amplifier SR570 was set as 50 nA/V to amplify the signal of the flexible sensor, and the output signal was recorded by an MP36 (Biopac, Goleta, CA, USA) collector for 10 s.

Heart sounds are caused by the closure of the valve. The cardiac cycle of a healthy adult is about 0.8 s, while the systolic time is 0.3 s and the diastolic time is 0.5 s. S1 is the heart sound signal formed by ventricular contraction, which makes the ventricular pressure exceed the atrial pressure, and the blood forces the atrioventricular valve to vibrate. The S1 sound pitch is low (around 40~60 Hz) but lasts for a long time (about 70–150 ms) with a high sound intensity. S2 marks the beginning of the ventricular diastole. This is the vibration caused by the outflow of blood from the ventricular, causing closure of the aortic and pulmonary valves. The S2 sound pitch is high (around 60–100 Hz) but lasts for a short time (about 60–120 ms) with a low sound intensity [51].

**Figure 9:**
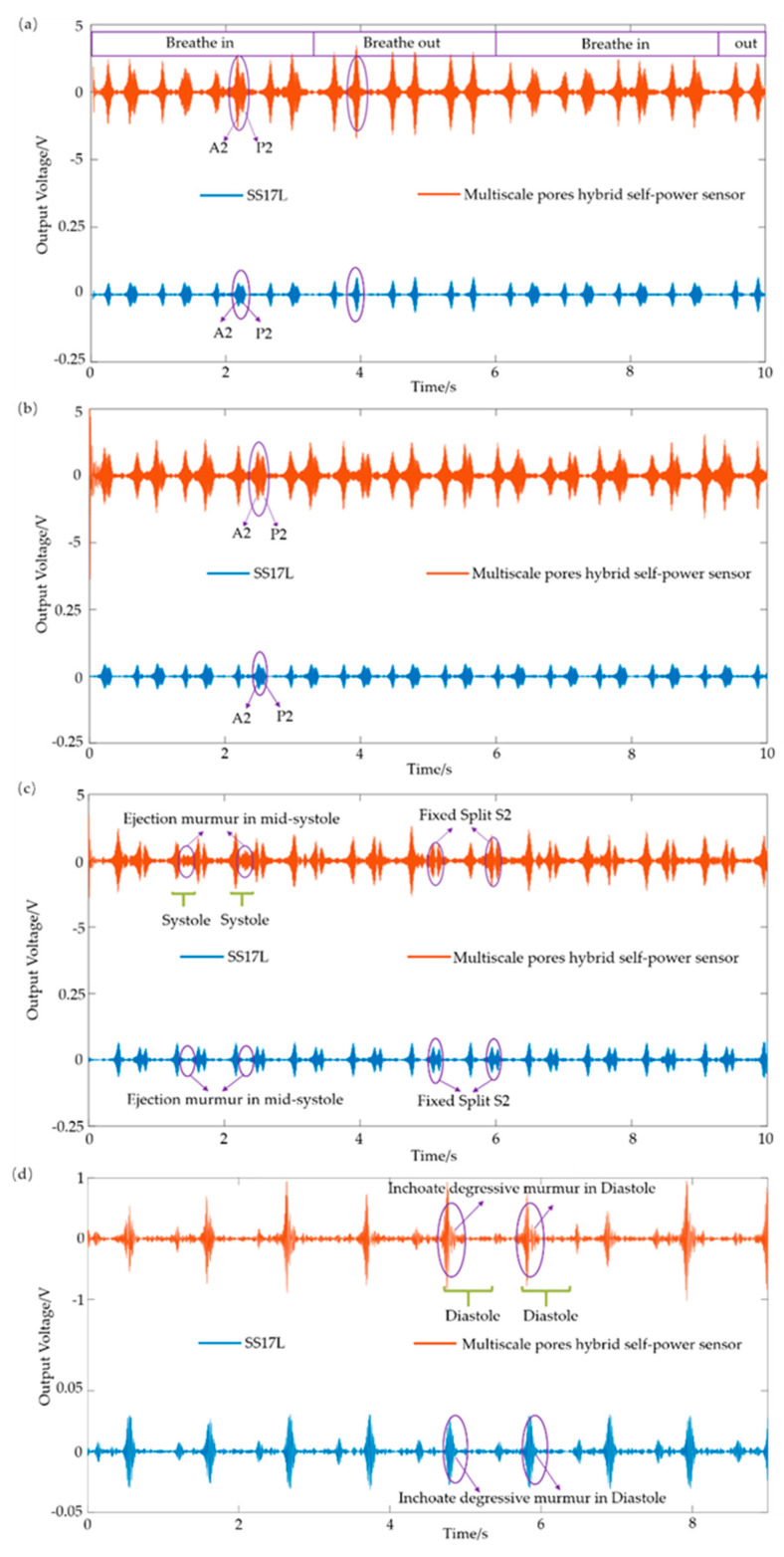
Schematic of pathological heart sound detection of SS17L (BIOPAC, Goleta, California, USA) and the proposed sensor. Representative features of (**a**) heart sound signal with a transient S2 split, (**b**) heart sound signal with a fixed S2 split, (**c**) heart sound signal of aortic regurgitation, and (**d**) heart sound signal of an atrial septal defect.

Figure 9a shows the heart disease signal of transient S2 splits. The time interval of the transient split is wider during inhalation, where the closure of the aortic valve (A2) and the pulmonary valve (P2) are clearly distinguished, and the time interval becomes narrow or indistinguishable during exhalation. Figure 9b shows the disease signal of fixed S2 splits. Patients with diseases such as pulmonary stenosis or heart failure show fixed S2 splits all the time, where A2 and P2 are clearly distinguished all the way. Figure 9c shows the detection results with the atrial septal defect heart disease. The disease is characterized by a mid-systolic ejection murmur accompanied by a fixed split of the S2 signal. The ejection murmur is caused by increased blood flow through the pulmonary valve, and the fixed split of S2 is caused by delayed closure of the valve. Figure 9d shows the features of the heart sound signals with aortic regurgitation disease, where the inchoate degressive murmur in the diastole is clearly distinguished. The results demonstrated that the flexible sensor had the same capability to distinguish the physiological and pathological heart sound signals as the commercial device.

### 4.4. Heart Sound Detection

**Figure 10:**
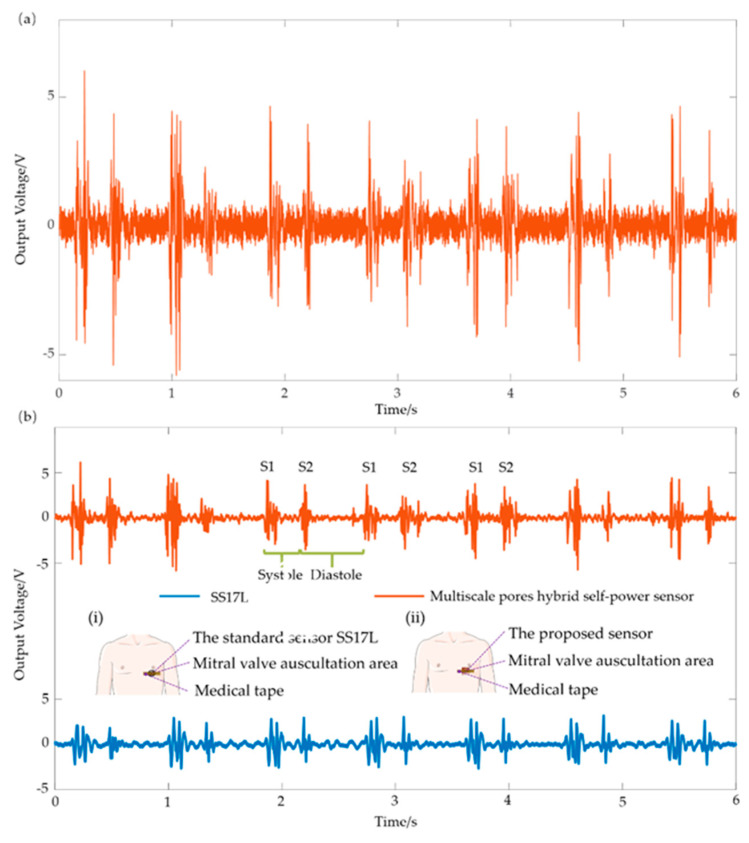
Schematic of heart sound detection in the human body. (**a**) Original signal of the heart sound signal; (**b**) heart sound signals after treatment with a filter. (**i**) Schematic drawing of the attachment of the standard sensor SS17L (BIOPAC, Goleta, CA, USA); (**ii**) schematic drawing of the attachment of the proposed flexible sensor.

In the process of detecting real heart sounds in the human body, the flexible sensor is directly attached to the skin. There are several areas for the auscultation of heart sounds, for example, the aortic valve area, the tricuspid valve, the pulmonary valve area and the mitral valve [54]. In the mitral valve auscultation area, the heartbeat is the loudest and clearest, where a high-quality heart sound signal can be collected. The flexible sensor was attached with medical tape to the mitral valve area of a volunteer (a 25-year-old healthy male without heart disease) to collect the heart sound signal. During the test, the subject was comfortably seated on a chair with a backrest support. Figure 10a shows the original signal of the human body. The original signal was filtered using the filter designer FDATOOL plugin of MATLAB. The sampling rate was set as 2000 Hz, and the response type was set as a bandpass with 55–95 Hz. Figure 10b shows the heart sound signal collected by the standard sensor SS17L and the self-made sensor at the same time. The attachment method and the measuring position of the standard sensor and the proposed flexible sensor are shown in the illustrations in Figure 10b(i,ii). It can be seen that the two heart sound features, S1 and S2, are basically the same between the self-made sensor and the commercial standard sensor. The heart sound detected by the self-made sensor had a systolic and diastolic time of 0.358 s and 0.515 s respectively, as well as a cardiac cycle of 0.873 s, while the standard sensor had a systolic and diastolic time of 0.331 s and 0.553 s, respectively, as well as a cardiac cycle of 0.884 s. Therefore, the error of the sensor in the time domain was about 1%, belonging to an acceptable range.

**Figure 11:**
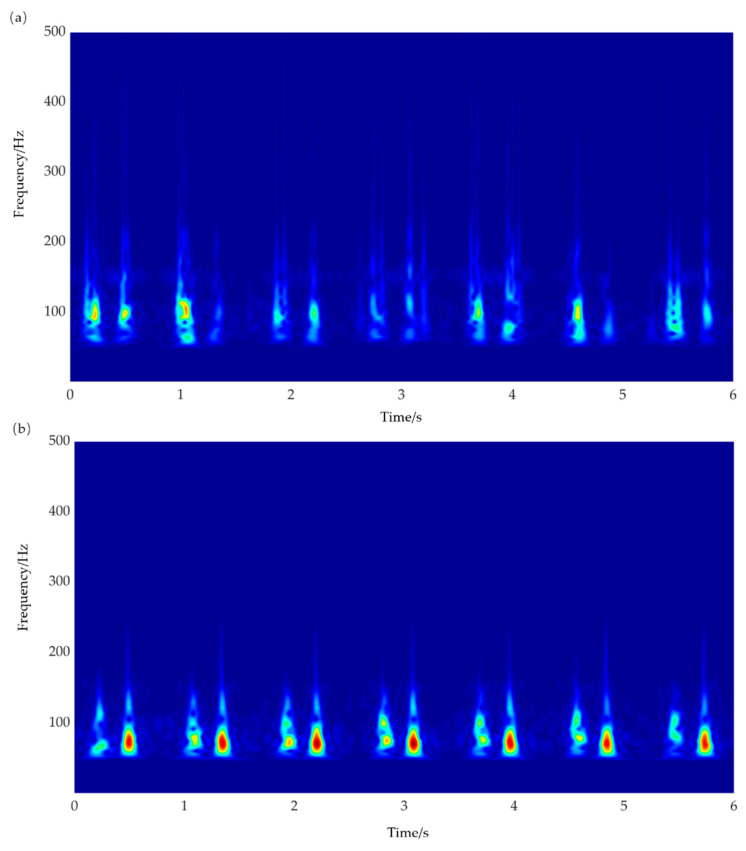
Schematic of time-frequency diagrams after wavelet transformation. (**a**) Time-frequency diagram of the proposed flexible sensor; (**b**) time-frequency diagram of the standard SS17L sensor.

Wavelet transformation was applied to the filtered signals, and the time-frequency diagrams are shown in Figure 11a,b. The signals detected by the proposed flexible sensor and the standard SS17L sensor were distributed mainly at 100 Hz, showing that low frequency interference was eliminated by filtering. The deficiency is that the low-frequency response of the sensor was not ideal, which needs to be improved in the future.

The flexible sensor offers primary verification of heart sound detection. Compared with other commercial electronic stethoscopes, this sensor is flexible with biological safety due to the materials used, and with electrical safety due to the self-powered characteristics, which provide the prospect of wearable heart sound detection by this sensor.

## 5. Conclusions

This paper proposes a novel flexible sensor with bi-nano-microscale pores and dual ferro-electret hybrid self-powering. Electrospinning was used to fabricate the piezoelectric film. Piezoelectric materials fabricated by electrospinning had dipoles directly and could be obtained in large areas during one-time rolling, which makes it possible to meet the requirements of mass production. The multiscale pore structure of the piezoelectric film was realized by the electrospinning process and nanoparticle etching with concentrated HCl. The piezoelectric film was then encapsulated by PDMS film, which provided an airtight space for corona polarization. Corona poling provided the device with real gas phase space charges for the sensor. The test results indicated that multiscale pores and corona poling significantly improved the sensitivity of the sensor. A piezoelectric coefficient of 3312 pC/N was achieved, which is about 10 times larger than that of the piezoelectric coefficients of comment piezoelectric polymers. The comparison experiment showed that the pathological heart sounds detected by the flexible sensor were consistent with the commercial device. The human heart sound detection experiment demonstrated the potential application of the flexible sensor for wearable heart sound detection. In addition to the novelty of the sensor’s configuration and fabrication, the proposed flexible sensor has the advantages of flexibility, low production cost and a short processing time compared with the existing heart sound detection sensors.

## Data Availability

The data presented in this study are available on request from the corresponding author.
